# A nisin-inducible chromosomal gene expression system based on ICE Tn*5253* of *Streptococcus pneumoniae*, transferable among streptococci and enterococci

**DOI:** 10.1007/s11274-024-04124-6

**Published:** 2024-09-12

**Authors:** Mariana Tirziu, Lorenzo Colombini, Maria Alfreda Stincarelli, Anna Maria Cuppone, Elisa Lazzeri, Francesco Santoro, Gianni Pozzi, Francesco Iannelli

**Affiliations:** Laboratory of Molecular Microbiology and Biotechnology (LAMMB), Department of Medical Biotechnologies, University of SienaViale Bracci, Policlinico Le Scotte, V Lotto I Piano 53100 Siena, Italy

**Keywords:** Integrative conjugative element (ICE), Tn*5253*, Tn*5251*, Tn*916*, Insertion vectors, Host-vector system, Gene expression, Nisin, *Streptococcus pneumoniae*, *Lactococcus lactis*

## Abstract

The present work reports the development and validation of a chromosomal expression system in *Streptococcus pneumoniae* which permits gene expression under the control of *Lactococcus lactis* lantibiotic nisin. The system is based on the integrative and conjugative element (ICE) Tn*5253* of *S. pneumoniae* capable of site-specific chromosomal integration and conjugal transfer to a variety of bacterial species. We constructed an insertion vector that integrates in Tn*5251*, an ICE contained in Tn*5253*, which carries the tetracycline resistance *tet*(M) gene. The vector contains the *nisRK* regulatory system operon, the *L. lactis* nisin inducible promoter P*nisA* upstream of a multiple cloning site for target DNA insertion, and is flanked by two DNA regions of Tn*5251* which drive homologous recombination in ICE Tn*5253*. For system evaluation, the *emm6.1::ha1* fusion gene was cloned and integrated into the chromosome of the Tn*5253*-carrying pneumococcal strain FR24 by transformation. This gene encodes a fusion protein containing the signal peptide, the 122 N-terminal and the 140 C-terminal aa of the *Streptococcus pyogenes* M6 surface protein joined to the HA1 subunit of the influenza virus A hemagglutinin. Quantitative RT-PCR analysis carried out on total RNA purified from nisin treated and untreated cultures showed an increase in *emm6.1::ha1* transcript copy number with growing nisin concentration. The expression of M6-HA1 protein was detected by Western blot and quantified by Dot blot, while Flow cytometry analysis confirmed the presence on the pneumococcal surface. Recombinant ICE Tn*5253*::[*nisRK*]-[*emm6.1::ha1*] containing the nisin-inducible expression system was successfully transferred by conjugation in different streptococcal species including *Streptococcus gordonii*, *S. pyogenes*,* Streptococcus agalactiae* and *Enterococcus faecalis*. As for *S. pneumoniae*, the *emm6.1::ha1* transcript copy number and the amount of M6-HA1 protein produced correlated with the nisin concentration used for induction in all investigated bacterial hosts. We demonstrated that this host-vector expression system is stably integrated as a single copy within the bacterial chromosome, is transferable to both transformable and non transformable bacterial species, and allows fine tuning of protein expression modulated by nisin concentration. These characteristics make our system suitable for a wide range of applications including complementation assays, physiological studies, host-pathogen interaction studies.

**Supplementary Information:**

The online version contains supplementary material available at 10.1007/s11274-024-04124-6.

## Introduction

Integrative and Conjugative Elements (ICEs) are Mobile Genetic Elements capable of stable integration into bacterial genome and intercellular transposition to a new genome by conjugal transfer to a broad range of hosts (Mullany et al. 2002). These characteristics make ICEs suitable tools for genetic manipulation of a large variety of transformable and non-transformable bacteria (Pozzi et al. 1988; Manganelli et al. 1998). ICEs can be used for introduction of heterologous DNA into recipient hosts, for reintroduction of in vitro mutagenized DNA in the original host, physiological studies, and complementation assays (Smith and Clewell 1984; Choi, Kyoung-Hee and Kim, Kang-Ju 2009;). ICE Tn*5253* (formerly called Ω6001) is a composite element found in *Streptococcus pneumoniae* BM6001 (Dang-Van et al. 1978; Colombini et al. 2023a) that contains Tn*5251*, an ICE essentially identical to the *Enterococcus faecalis* Tn*916* carrying the tetracycline resistance gene *tet*(M) (Provvedi et al. 1996; Santoro et al. 2010, 2014; Iannelli et al. 2014). Tn*5253* transfers in different bacterial species with a frequency of conjugation up to 6.7 × 10^− 3^ transconjugants per donor (Santoro et al. 2010, 2018). Chromosomal integration of Tn*5253* occurs downstream of a conserved 11-bp sequence of the essential *rbgA* gene in *S. pneumoniae* and in other bacterial species (Santoro et al. 2021). The insertion vector pDP36 was designed to integrate heterologous DNA into the chromosome of streptococci carrying Tn*5253* and the resulting recombinant ICE was transferred to streptococci and enterococci by conjugation (Pozzi et al. 1988; Oggioni and Pozzi 1990). Vector pSMB47, a derivative of pDP36, carrying two non-contiguous DNA fragments of Tn*5251* was constructed and used to introduce heterologous DNA in *Bacillus subtilis* and *E. faecalis* strains carrying Tn*916* (Manganelli et al. 1998). A host-vector system for construction, chromosomal integration, and expression of translational-fusions with the *Streptococcus pyogenes emm6.1* gene, encoding the surface protein M6, was developed and used for surface display of several heterologous proteins in *Streptococcus gordonii* (Pozzi et al. 1992a, b; Oggioni and Pozzi 1996; Oggioni et al. 1999). The system is based on an insertion vector which allows for chromosomal integration of fusion genes under the control of a strong constitutive promoter in a specifically engineered *S. gordonii* transformation recipient. *S. pneumoniae* is an important human pathogen, naturally competent for genetic transformation, easy to genetically manipulate, thus representing a model for the study of bacterial genetics and pathogenesis (Santoro et al. 2019). Several genetic tools have been developed to manipulate *S. pneumoniae*, among these controlled gene expression systems represent a powerful tool to study pneumococcal physiology. The *S. pneumoniae* Chromosomal Expression Platform (CEP), permits expression of a target gene under the control of a maltose-inducible promoter (Guiral et al. 2006a, b). This system, located in a silent transcriptional site and based on a preassembled insertion vector, was used for controlled expression of the luciferase gene and the competence *comDE* operon. The Pneumococcal Engineering Platform (PEP) is based on insertion vectors carrying an IPTG-inducible promoter, able to integrate in the majority of *S. pneumoniae* genomes. This system was efficiently utilized to express newly described fluorescent proteins (Keller et al. 2019). The nisin-controlled gene expression system (NICE), based on nisin biosynthesis autoregulation, was developed in *L. lactis* and used to express genes of different origins in different hosts including *S. pneumoniae*. The system relies on target gene cloning downstream of the nisin inducible P*nisA* promoter via the two-component system *nisRK* encoding the histidine kinase NisK and the response regulator NisR (Kuipers et al. 1993, 1995; De Vos et al. 1995; Zhou et al. 2006). In this study, we developed a chromosomally stable host-vector expression system, inducible by nisin in *S. pneumoniae*. The system is constituted by an insertion vector designed to integrate in the pneumococcal ICE Tn*5253* and is therefore transferable by conjugation to a broad range of bacterial species, making the system suitable for a wide range of applications including complementation assays, physiological studies, host-pathogen interaction studies.

## Materials and methods

### Bacterial strains, growth conditions, and minimal inhibitory concentration (MIC) determination

Bacterial strains used in this work and their relevant properties are reported in Table 1. Streptococci and enterococci strains were routinely grown at 37°C in tryptic soy broth (TSB) or in tryptic soy agar (BD Difco) supplemented with 3% horse blood and where appropriate with antibiotics (Santoro et al. 2010; Iannelli et al. 2021). *Escherichia coli* was grown in Luria-Bertani broth (LB) or LB supplemented with 1.5% agar (LBA). When required, 20 µg/ml chloramphenicol was added to both LB and LBA media. Bacteria for Western blot, Dot blot, Flow cytometry and Immunofluorescence microscopy analysis were grown in TSB without dextrose (BD Difco) until the late exponential phase (OD_590_ = 0.3, corresponding to approximately 10^6^ CFU/ml). Nisin for induction was extracted as already described (Xiao et al. 2010). Briefly, 100 mg of 2.5% nisin (Sigma-Aldrich) was resuspended in 50 ml of 50% ethanol in distilled water, stirred for 2 h before centrifugation at 1,500 x *g* for 5 min, and supernatant was then collected and stored at -20°C. Nisin sensitivity was determined by MIC assays as already described (Fox et al. 2021). Briefly, bacteria were grown in TSB until reaching the exponential phase (OD_590_ = 0.3, corresponding to approximately 10^8^ CFU/ml), then culture aliquots were diluted 1:100 in TSB (10^6^ CFU/ml) and 100 µl were added to a 96-wells microplate containing 100 µl of serial twofold dilutions of nisin, reaching a final concentration of 5 × 10^5^ CFU/ml in each well. Plates were incubated at 37°C and visually analyzed after 18 h. Bacterial growth was assessed using the microplate ELISA reader VERSAmax (Molecular Devices). The nisin MIC value for the *S. pneumoniae* recombinant FR372 strain was 1 µg/ml. For nisin treatment, bacterial cultures grown in TSB until mid-exponential phase (OD_590_ = 0.3) were split in 5 different aliquots of which 4 were treated with 3-fold serially diluted nisin concentrations (from 27 to 1 ng/ml). Cultures were incubated at 37°C for 60 min, then bacterial cells were harvested by centrifugation at 10,000 x *g* at 4°C for 5 min and stored at -70°C in phosphate-buffered saline (PBS) containing 10% glycerol.

**Table 1:** Bacterial strains

Strain	Relevant properties^a^	Origin (Reference)
Rx1	*S. pneumoniae* unencapsulated standard transformation recipient	(Cuppone et al. 2021)
FP10	Rx1 competence deficient derivative, Δ*comC*, *str-41*; Cm^R^, Sm^R^	(Santoro et al. 2010)
FR24	FP10 derivative carrying Tn*5253*, Δ*comC*, *str-41*, *tet*(M); Cm^R^, Sm^R^, Tc^R^	(Iannelli et al. 2018)
V288	*S. gordonii* Challis, clinical isolate	(Macrina et al. 1980; Vickerman et al. 2007)
GP204	V288 derivative, *str-204*; Sm^R^	(Pozzi et al. 1988)
GP1295	V288 derivative carrying the *emm6.1::aph*III locus for gene cloning, *str-204*, *aph*III; Sm^R^, Km^R^	(Oggioni and Pozzi 1996; Falcone et al. 2006)
FR201	GP1295 derivative carrying the fusion gene *emm6.1::ha1*, *str-204*, *erm*(C); Sm^R^, Em^R^	(Iannelli unpublished)
SF370	*S. pyogenes* serotype M1	(Ferretti et al. 2001)
H36B	*S. agalactiae* serotype Ib	(Tettelin et al. 2005)
JH2-2	*E. faecalis*; Fus^R^, Rif^R^	(Jacob and Hobbs 1974)
DH5α	*E. coli* standard transformation recipient; Amp^R^	(Grant et al. 1990)
GP394	DH5α derivative carrying insertion vector pSMB47, *lacZ*,* erm*(B), *cat*; Amp^R^, Em^R^, Cm^R^	(Manganelli et al. 1998)
FR369	DH5α derivative carrying pMBT-5, *erm*(B), *cat*; Amp^R^, Em^R^, Cm^R^	This study
FR370	DH5α derivative carrying pMBT-6*erm*(B), *cat*; Amp^R^, Em^R^, Cm^R^	This study
FR371	FR24 derivative carrying Tn*5253*::[*nisRK*] (by transformation with pMBT-5), Δ*comC*; *str-41*, Δ*tet*(M), *erm*(B), *cat*; Sm^R^, Em^R^, Cm^R^	This study
FR386	GP204 derivative carrying Tn*5253*::[*nisRK*] (by conjugation with FR371), *str-204*, Δ*tet*(M), *erm*(B), *cat*; Sm^R^, Em^R^, Cm^R^	This study
FR372	FR24 derivative carrying Tn*5253*::[*nisRK*]*-*[*emm6.1::ha1*] (by transformation with pMBT-6), Δ*comC*; *str-41*, Δ*tet*(M), *erm*(B), *cat*; Sm^R^, Em^R^, Cm^R^	This study
FR387	GP204 derivative carrying Tn*5253*::[*nisRK*]*-*[*emm6.1::ha1*] (by conjugation with FR372), *str-204*, Δ*tet*(M), *erm*(B), *cat*; Sm^R^, Em^R^, Cm^R^	This study
FR389	SF370 derivative carrying Tn*5253*::[*nisRK*]*-*[*emm6.1::ha1*] (by conjugation with FR372), Δ*tet*(M), *erm*(B), *cat*; Em^R^, Cm^R^	This study
FR388	H36B derivative carrying Tn*5253*::[*nisRK*]*-*[*emm6.1::ha1*] (by conjugation with FR372), Δ*tet*(M), *erm*(B), *cat*; Em^R^, Cm^R^	This study
FR390	JH2-2 derivative carrying Tn*5253*::[*nisRK*]*-*[*emm6.1::ha1*] (by conjugation with FR372), Δ*tet*(M), *erm*(B), *cat*; Fus^R^, Rif^R^, Em^R^, Cm^R^	This study

^a^*comC* encodes the competence stimulating peptide precursor (Pozzi et al. 1996; Iannelli et al. 2005); *emm6.1* encodes the *S. pyogenes* M6 surface protein (Fischetti et al. 1988); *aph*III confers resistance to kanamycin; *erm*(B) and *erm*(C) confer resistance to erythromycin (Cassone et al. 2006); *str-41* and *str-204* indicate point mutations conferring resistance to streptomycin; Cm, chloramphenicol; Sm, streptomycin; Tc, tetracycline; Km, kanamycin; Em, erythromycin; Amp, ampicillin; Fus, fusidic acid; Rif, rifampicin

### Vectors construction

**Fig. 1:**
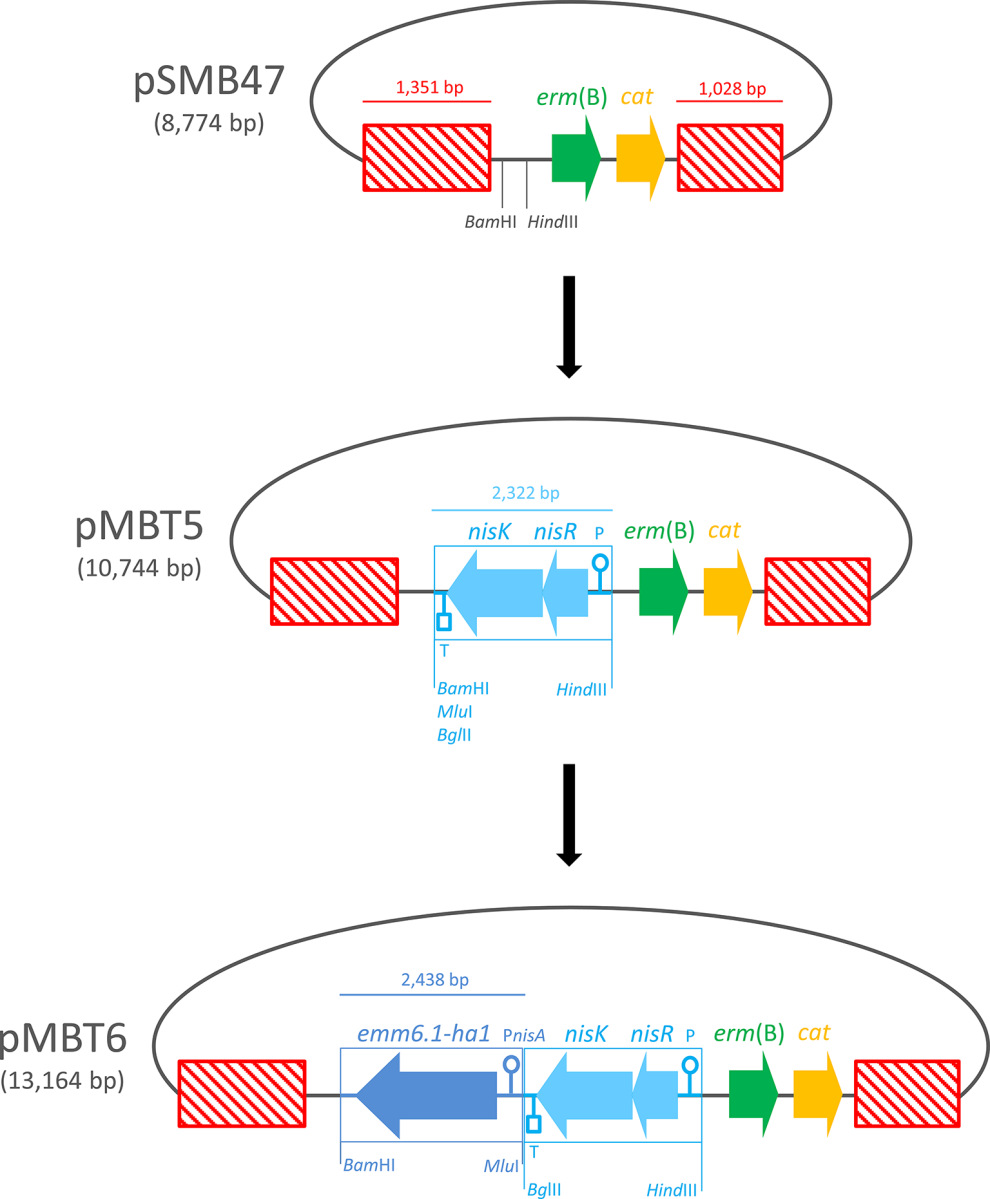
Schematic representation of pMBT6 vector construction. Insertion vector pSMB47 contains: (i) the chloramphenicol resistance *cat* and erythromycin resistance *erm* (B) genes allowing for selection in *E. coli* and gram-positive bacteria, respectively; and (ii) two non-contiguous fragments of the ICE Tn*5251* (striped red boxes) driving integration in the element. The 2,322-bp synthetic construct containing the *L. lactis nisRK* two-component regulatory system was inserted in pSMB47 between the *Bam*HI and *Hind*III restriction sites producing pMBT5. Then, the 2,438-bp synthetic construct containing the *emm6.1::ha1* fusion gene was inserted in pMBT5 between the *Bam*HI and *Mlu*I restriction sites producing pMBT6. ORFs and their direction of transcription are represented by arrows and indicated with their names. Promoters, terminator and restriction sites are reported. Figure is not drawn to scale

Standard molecular cloning procedures were used. Briefly, the restriction reactions mixture contained, in a final volume of 50 µl, 1× buffer, 1 U enzyme, and 1 µg plasmid DNA. Mixture was incubated at 37°C for 60 min, then enzyme was inactivated at 65°C for 20 min. If necessary, mixture was purified with the *Quick*-DNA purification kit according to manufacturer’s instructions (Zymo Research). Digested DNA was visualized and quantified by both electrophoretic and spectrophotometric analyses. The sequence of vectors was verified by PCR followed by Sanger sequencing as reported (Iannelli et al. 1998). Insertion vector pSMB47 (GenBank no. U69267) contains the *cat* and *erm*(B) genes conferring resistance to chloramphenicol and erythromycin allowing for selection in *E. coli* and gram-positive bacteria, respectively (Fig. 1) (Manganelli et al. 1998). The vector contains two non-contiguous fragments of the ICE Tn*5251*. The fragments are 1,351 bp and 1,028 bp in length and correspond to nucleotides (nts) 4,080 to 5,430 and 6,980 to 8,007 of Tn*5251* sequence (GenBank no. FJ711160.1). Vector pSMB47 was purified from *E. coli* GP394, digested with *Bam*HI and *Hind*III restriction enzymes and ligated to a 2,322-bp synthetic DNA fragment containing the *L. lactis nisRK* operon (2,227 bp, nts 305,189 to 307,415 GenBank no. CP002094) joined to the *S. pneumoniae dexB* terminator (71 bp, nts 380 to 450 GenBank no. AF026471.3). The fragment, customized by DNA2.0 ATUM company, is flanked by *Hind*III on one end and by *Bam*HI, *Mlu*I, *Bgl*II on the other end. The resulting vector pMBT5 purified from the recombinant *E. coli* strain FR369, was digested with *Bam*HI and *Mlu*I and ligated to a second 2,438-bp synthetic DNA fragment containing the fusion gene *emm6.1::ha1* (*emm6.1* GenBank no. M11338; *ha1* GenBank no. FJ969540) joined to the 277-bp DNA fragment located upstream the start codon of the *L. lactis nisA* nisin coding sequence containing the P*nisA* promoter sequence (nts 1 to 277, GenBank no. AF465351.1). The fragment, customized by DNA2.0 ATUM company, is flanked by *Bam*HI on one end and *Mlu*I, *Bgl*II on the other end. The *emm6.1::ha1* contains: (i) 126 nts encoding the 42-aa signal peptide of the *Streptococcus pyogenes* M6 surface protein; (ii) the 366 nts encoding the first N-terminal 122 aa of mature M6; (iii) 966 nts encoding the first N-terminal 322 aa of the HA1 subunit of the influenza virus A hemagglutinin (HA); (iv) 420 nts encoding the last C-terminal 140 aa of mature M6 containing the cell wall anchor domain; (v) 271 nts containing the 80 nts located downstream the *emm6.1* CDS. The resulting vector pMBT6 was transformed into *E. coli* competent cells and the representative strain FR370 was used for further experiments.

### Preparation of competent cells and transformation

*S. pneumoniae* competent cells preparation and transformation were carried out as reported (Iannelli and Pozzi 2004). Frozen pneumococcal competent cells were thawed and incubated at 37°C for 10 min in the presence of 100 ng/ml competence stimulating peptide (competence induction). Then transforming DNA was added at a final concentration of 1 µg/ml and the mixture was incubated at 30°C for 20 min (transformation reaction). Prior to transformation, DNA vectors pMBT5 and pMBT6 were linearized with *Kpn*I and *Sac*I, respectively. Selection of recombinant strains was obtained by a multilayer plating procedure (Iannelli et al. 2021). The frequency of transformation was 3.4 × 10^− 2^ transformants/total CFU and 5.4 × 10^− 2^ transformants/total CFU when pMBT5 or pMBT6 was used as donor DNA, respectively. *E. coli* competent cells were obtained as follows: an overnight culture was diluted 100-fold in 200 ml of LB medium and incubated at 37°C with shaking at 180 RPM; upon reaching an OD_590_ = 0.6, cells were centrifuged at 5,000 x *g* for 10 min at 4°C in 50-ml tubes; each pellet was gently resuspended in 25 ml ice-cold 0.1 M MgCl_2_ solution and centrifuged at 5,000 x *g* at 4°C for 10 min; finally, the cell pellet was resuspended in 5 ml of an ice-cold 0.1 M MgCl_2_ solution supplemented with 10% glycerol, aliquoted and frozen at -70°C (Chang et al. 2017). Transformation of *E. coli* competent cells was performed as follows: 0.2 ml of competent cells containing the transforming DNA at a final concentration of 0.1 µg/ml were incubated in an ice bath for 10 min swirling gently every 2 min, heat shocked at 42°C for 2 min and placed back on ice for 5 min; volume was adjusted to 1 ml with LB containing 10% glycerol, the mixture was incubated at 37°C with shaking at 180 RPM for 180 min and then frozen at -70°C. Recombinant strains were selected by spreading 0.01 ml of the transformation mixture onto LBA plates containing the appropriate antibiotic.

### Mating assays

The protocol for plate mating experiments was already described (Iannelli et al. 2021). Briefly, donor and recipient cells were grown separately in TSB in the presence of the appropriate antibiotic at 37°C, until the end of the exponential phase (OD_590_ = 0.8). Cells were mixed at a 1:10 ratio, harvested by centrifugation at 3,000 x *g* for 15 min, resuspended in 0.1 ml of TSB and plated on TSA plates enriched with 5% horse blood. After 4 h of incubation in 5% CO2 at 37°C, cells were harvested by scraping the plate with a sterile swab and resuspended in 1 ml TSB containing 10% glycerol. Transconjugants were selected with a multilayer plating procedure.

### DNA and RNA purification

Plasmid DNA purification was carried out using the NucleoSpin Plasmid Kit (Macherey-Nagel) following manufacturer’s instructions. Briefly, *E. coli* cells were cultured in LB medium until reaching OD_590_ = 3.0, harvested by centrifugation, lysed and loaded on the column. DNA was eluted in 30 µl of 5 mM Tris/HCl pH 8.5. Purified DNA was quantified by both electrophoretic and spectrophotometric analyses. Total RNA was purified from bacterial cultures using the NucleoSpin RNA Isolation Kit (Macherey-Nagel) according to manufacturer’s instructions. Purified RNA was subjected to amplification grade DNase I treatment (AMPD1, Sigma-Aldrich). The 10 µL reaction mixture was in 1× reaction buffer, containing 1 unit of DNase I and 500 ng of RNA preparation, and was incubated at 37°C for 15 min. EDTA stop solution was added at a final concentration of 5 mM, while DNase I was inactivated by heating at 70°C for 10 min. Agilent 2100 Bioanalyzer (Agilent Technologies) apparatus and the Agilent RNA 6000 Nano Kit (Agilent Technologies) were used to evaluate RNA integrity.

### Reverse transcription and quantitative real-time PCR

Reverse transcription for cDNA synthesis was carried out using the Transcriptor First Strand cDNA Synthesis Kit (Roche) essentially following the kit manufacturer’s instructions. Briefly, a mixture containing, in a final volume of 13 µl, 60 µM random hexamer primers and 0.05 µg of total RNA was incubated at 65°C for 10 min (denaturation step) and placed on ice. Then, a 7 µl mixture containing 1× Transcriptor Reverse Transcriptase Reaction buffer, 20 U Protector RNase Inhibitor, 1 mM Deoxynucleotide Mix and 10 U Transcriptor Reverse Transcriptase, was added. The 20 µl mixture was incubated at 25°C for 10 min, followed by 60 min at 50°C, inactivated at 85°C for 5 min and finally cooled on ice. Real-time PCR mixture contained, in a final volume of 20 µl, 1× KAPA SYBR FAST qPCR reaction mix, 5 pmol of each primer and 1 µl of cDNA. Thermal profile was an initial 3 min denaturation step at 95°C followed by 40 cycles of repeated denaturation (0 s at 95°C), annealing (20 s at 60°C), and polymerization (45 s at 72°C). The temperature transition rate was 20°C/s in the denaturation and annealing step and 5°C/s in the polymerization step. Oligonucleotide primers targeting the *emm6.1::ha1* gene were IF994 (5’-CCGAACCACGATAGCGACAA-3’) and IF995 (5’-CCACAGGACCAGGACTTCTT-3’). Serial dilutions of the *S. pneumoniae* FR24 chromosomal DNA with known concentration were used to build a standard curve for the *gyrB* gene by plotting the threshold cycle against the number of chromosome copies (Lazzeri et al. 2012; Santoro et al. 2023). This standard curve was recalled in the instrument software to standardize the *emm6.1::ha1* transcript copy number. To discriminate desired amplification products from primer-dimer products, the melting curves were analysed. As an internal expression control we used the housekeeping *gyrB* gene.

### Nanopore sequencing

High-molecular-weight genomic DNA was purified using a raffinose-based method as reported (Teodori et al. 2021; Pinzauti et al. 2022b). Briefly, a bacterial cell-wall enzymatic digestion was performed followed by protoplast osmotic lysis and DNA purification with Sevag (chloroform-isoamyl alcohol, 24:1 [vol: vol]). Purified genomic DNA was size-selected with 0.5× AMPure XP beads (Beckman Coulter) and used for Nanopore sequencing libraries preparation with the SQK-LSK108 kit (Oxford Nanopore Technologies) following the manufacturer’s instructions. Sequencing runs were managed by a GridION X5 device (Oxford Nanopore Technologies) as previously described (Pinzauti et al. 2022a).

### Long PCR and sanger sequencing

Long PCRs and direct PCR sequencing were carried out as described (Iannelli et al. 1998). The primer pair IF394 (5’-GCTATAGTATAAGCCATACTT-3’) and IF668 (5’-GTTGTGATTGCCTTGTGGGT-3’) directed at the *tet*(M) and *orf13* (nts 4,387 - 4,407 and 7,092 - 7,111 of Tn*5251*, GenBank accession no. FJ711160.1), respectively, were used to amplify the integrated genetic construct, whereas already described primer pairs directed to Tn*5253* and chromosomal flanking regions (Iannelli et al. 2014; Santoro et al. 2018, 2021) were used to confirm structure and chromosomal integration of the recombinat Tn*5253*::[*nisRK*]-[*emm6::ha1*] element.

### Western blot analysis

A total of 10^7^ colony forming units (CFUs) was incubated at 37°C for 60 min in 200 µl of Protoplasting Buffer (20% Raffinose, 50 mM Tris-HCl pH 8.0, 50 mM EDTA) containing 4 mg/ml lysozyme (Colombini et al. 2023b). The protoplasts were sedimented by centrifugation at 5,000 x *g* for 5 min and the supernatant was collected. To concentrate the supernatant, 4 volumes of acetone were added and the mixture was incubated at -20°C for 60 min, then centrifuged at 15,000 x *g* for 10 min. The pellet was air-dried for 15 min and resuspended in 50 µl of 10 mM Tris, 1 mM EDTA, 2% SDS buffer. Cell wall extract was quantified using the Qubit Protein Assay Kit (ThermoFisher), according to manufacturer’s instructions, washed in ice-cold PBS and resuspended in 1× NuPAGE LDS Sample Buffer and 1× NuPAGE Sample Reducing Agent, incubated at 70°C for 10 min, then separated on 4–12% NuPAGE Bis-Tris Protein Gel on a XCell SureLock Mini-Cell device following manufacturer’s instructions (ThermoFisher). The gel was dry transferred onto nitrocellulose membrane (ThermoFisher) using the iBlot Dry Blotting System apparatus (ThermoFisher) and blocked with 1× Tris Buffered-Saline (TBS) (150mM NaCl, 20 mM Tris-Cl, pH 7.5) containing 0.05% Tween20 (Sigma-Aldrich) and 5% nonfat dry milk (Applichem) for 2 h at room temperature (RT) on a rocking platform. The membrane was incubated at 4°C overnight in the presence of anti-M6 rabbit serum, diluted 1:1,000 in 1× TBS, 0.05% Tween20, 3% milk. The membrane was washed 3 times for 10 min in 1× TBS, 0.05% Tween20, and then incubated for 60 min at RT in the presence of goat anti-rabbit IgG Alkaline-Phosphatase conjugated antibody (Sigma-Aldrich) diluted 1:10,000 in 1× TBS, 0.05% Tween20, 3% milk. The membrane was washed twice in 1× TBS 0.05% Tween20 3% milk for 3 min and once in 1× TBS for 15 min. Proteins were detected using the colorimetric detection of Alkaline-Phosphatase labeled molecules method. Briefly, 1,65 mg/ml 5-bromo-4-chloro-3-indolyphosphate-p-toluidine (BCIP) salt and 3.3 mg/ml nitro-blue tetrazolium chloride (NBT) chromogen (Sigma-Aldrich) were added to 100 mM Tris, 100 mM NaCl and 5 mM MgCl_2_ pH 9.5 working solution, according to standard procedures. The membrane was incubated in this substrate buffer until color development appeared and the reaction was stopped washing the membrane with distilled water.

### Dot blot analysis

Bacterial cells were spotted onto a nitrocellulose membrane with a Bio-Dot microfiltration blotting apparatus (Bio-Rad) using a vacuum-manifold procedure. Sample spotting was performed in two-fold serial dilutions from 1 × 10^7^ to 7.8 × 10^4^ CFU. Uninduced recombinant strains and their parental strains were spotted as controls. The membrane was dried at 80°C and blocked with TBS containing 0.05% Tween20 and 5% nonfat dry milk for 2 h at RT, followed by an overnight incubation at 4°C with rabbit anti-HA1 antibody (Sino Biological) diluted 1:1,000 in 1× TBS, 0.05% Tween20, 3% milk. The membrane was washed 3 times for 3 min with 1× TBS, 0.05% Tween20 and incubated for 60 min at RT with goat anti-rabbit IgG Alkaline-Phosphatase conjugated antibody (Sigma-Aldrich) diluted 1:10,000 in 1× TBS, 0.05% Tween20, 3% milk. The membrane was washed again 3 times for 3 min with 1× TBS, 0.05% Tween20 and once with TBS, for 15 min. For standard curve building, known quantities of the purified HA1 (Sino Biological) protein ranging from 25 ng to 195 pg were used. Protein quantification was obtained by densitometric analysis using the ImageJ v.1.53e software.

### Flow cytometry

A total of 10^7^ CFUs were washed in ice cold PBS and blocked in PBS-BSA 2% at 37°C for 30 min. Next, cells were incubated with primary anti-HA1 antibody (Sino Biological, 11055-RM10) diluted 1:160 in PBS-BSA 2% at 4°C for an hour, washed twice in PBS and incubated with 1:160 diluted secondary, FITC-conjugated, anti-rabbit IgG antibody (Sigma-Aldrich) at 37°C for 20 min. Cells were washed in PBS and fixed with BD Cytofix/Cytoperm solution (Becton Dickinson) at 4°C for 15 min. Samples were finally resuspended in 300 µl of filtered PBS and analysed using FACS scan instrument (Becton Dickinson).

### Immunofluorescence microscopy

*S. pneumoniae* FR372 cells, induced with nisin 27 mg/ml or not induced, were grown until late expenential phase, washed and resuspended in PBS. A total of 10 µl, corresponding to approximately 10^6^ CFU, were spotted on a glass slide and air-dried. Bacteria were fixed for 5 min in methanol and washed in PBS. The slide was incubated at 4°C for 1 h in a humidified chamber with anti-M6 rabbit serum, diluted 1:25 in PBS-BSA 2%. Then, cells were washed three times with PBS and incubated with a fluorochrome FITC-conjugated goat anti-rabbit IgG secondary antibody (Sigma) at 37°C for 30 min in a humidified chamber. Slide were washed three times with PBS, DAPI II counterstain (Abbot) was added at a final concentration of 12.5 pg/ml, washed again three times with PBS, air-dried, and covered with cover slip. Bacteria were observed under a Leica 6500 fluorescence microscope (Leica) using a 100x oil objective. Images were captured with a CFTR6500 digital camera (Leica).

## Results

### Construction of a nisin-inducible chromosomal gene expression system based on ICE Tn*5253*

**Fig. 2:**
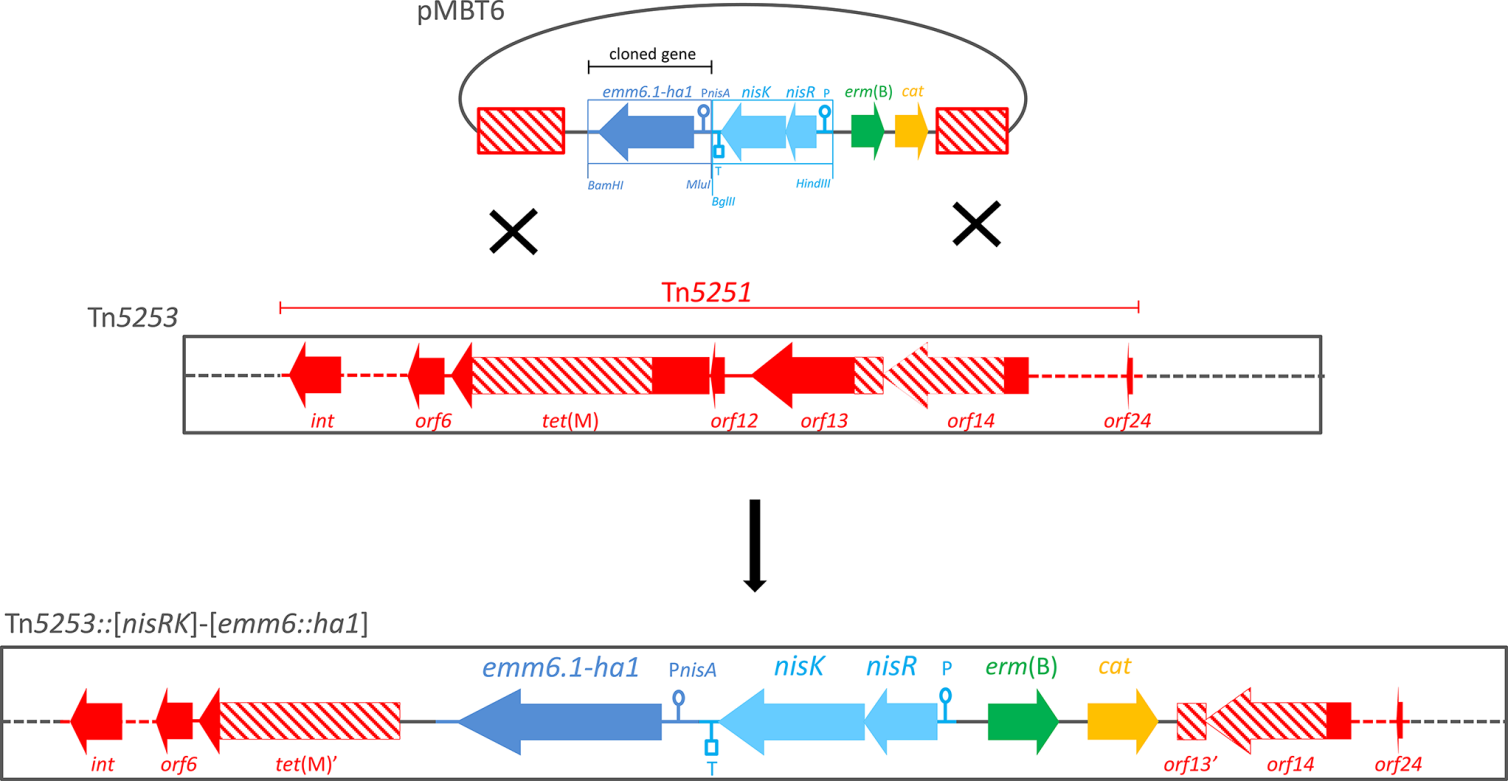
Schematic representation of the *S. pneumoniae* ICE Tn*5253*-based nisin-inducible gene expression system construction. The insertion vector pMBT6 contains: (i) the *L. lactis nisRK* two-component regulatory system; (ii) the *emm6.1::ha1* fusion gene under the control of the *L. lactis* P*nisA* promoter; and (iii) two flanking DNA fragments (striped red boxes) homologous to the ICE Tn*5251* which is integrated in the composite element Tn*5253.* Flanking fragments drive the integration of the genetic construct [*nisRK*]-[*emm6.1::ha1*] into Tn*5253* by double crossover recombination. ORFs and their direction of transcription are represented by arrows and indicated with their names. Promoters, terminator and restriction sites are reported. Figure is not drawn to scale

We constructed the insertion vector pMBT5, a derivative of pSMB47 (Manganelli et al. 1998), that integrates by double crossover recombination in Tn*5251*, an ICE contained in the composite element Tn*5253* and carrying the tetracycline resistance *tet*(M) gene (Fig. 1). The vector contains two Tn*5251* homologous regions to drive recombination, the *L. lactis* two-component regulatory system *nisRK* joined to the *S. pneumoniae dexB* terminator, and a multiple cloning site suitable for target gene insertion under control of the *L. lactis* nisin inducible promoter P*nisA* (Kuipers et al. 1993). Subsequently, a DNA fragment containing the fusion gene *emm6.1::ha1* joined to the P*nisA* promoter region was cloned in vector pMBT5, yielding vector pMBT6 (Fig. 1). Competent cells of *S. pneumoniae* FR24 containing Tn*5253* integrated into the chromosome, were transformed with a linearized pMBT6 vector (Fig. 2). Homologous recombination promotes integration of the genetic construct [*nisRK*]-[*emm6.1::ha1*] along with the *erm*(B) and *cat* resistance markers and the deletion of 1,549 bp including the first 480 nucleotides of the *tet*(M) coding sequence. Selection of recombinant strains was obtained by acquisition of erythromycin resistance and loss of tetracycline resistance. Genome sequence analysis of the representative erythromycin-resistant transformant FR372 confirmed the presence of the recombinant ICE Tn*5253*::[*nisRK*]-[*emm6.1::ha1*]. Genetic stability of the resistance phenotype was tested by growing pneumococcal cells in liquid medium without antibiotics for 220 generations. After plating on a non-selective medium, each of the 300 randomly picked colonies was resistant to erythromycin. PCR and sequencing carried out on 10 out 300 isolates confirmed that the genetic construct was intact and correctly integrated. The effect of the chromosomal integration of the construct on growth rate and on transformation frequency was investigated in the FR372 transformant and its FR24 parental strain. Both strains showed comparable duplication time (1 generation/hour) and transformation frequency (2% of total CFU).

### Evaluation of the nisin-inducible gene expression system in *Streptococcus pneumoniae*

**Fig. 3:**
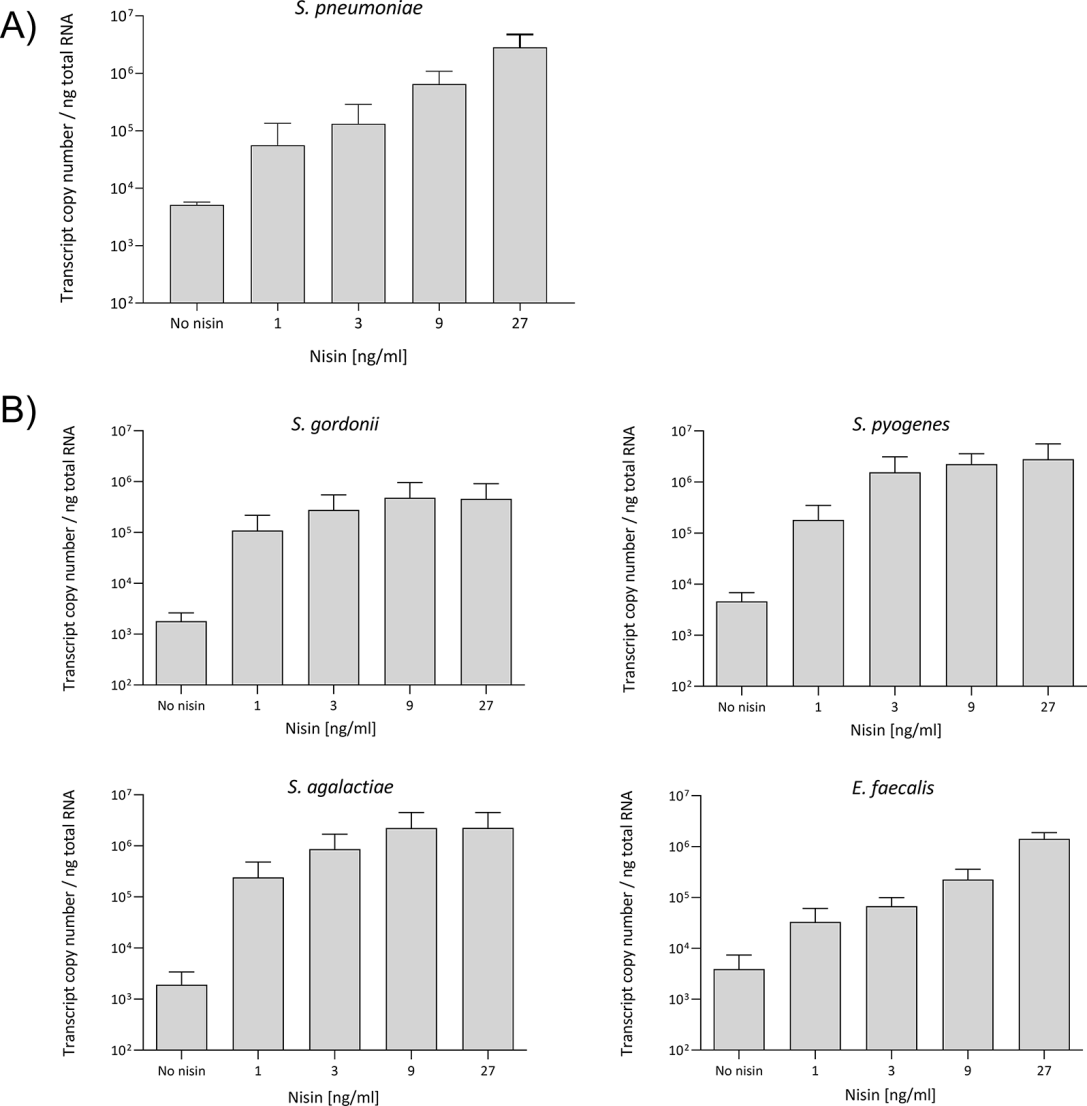
*Emm6.1::ha1* fusion gene expression upon nisin treatment in *S. pneumoniae* (**A**) and in other streptococci and enterococci (**B**). RT-qPCR was performed on total RNA purified from the recombinant strains using primers targeting the *emm6.1::ha1* fusion gene. Gene expression level was calculated as copy number per nanograms of total RNA. The *emm6.1::ha1* transcript copy number correlated with the nisin concentration used for induction ranging from 1 to 27 ng/ml. Results are reported as means and standard deviations resulting from 2 to 6 technical replicates from 2 independent experiments

Pneumococcal cultures of the FR372 recombinant strain in the exponential growth phase were treated with subinhibitory doses of nisin (from 1 to 27 ng/ml), and total RNA purified from treated and untreated control cultures was used as template for detection and quantification of the *emm6.1::ha1* transcript. A specific *emm6.1::ha1* transcript was found in both treated and untreated cultures, and the transcript copy number increased with the growing nisin concentration used for induction (Fig. 3A). The levels of gene expression ranged from 5.12 × 10^3^± 6.29 × 10^2^ to 2.83 × 10^6^ ± 1.90 × 10^6^ copies per nanograms of total RNA, obtained from either untreated or nisin treated (27 ng/ml) cultures.

### Detection and quantification of the induced protein production in *S. pneumoniae*

**Fig. 4:**
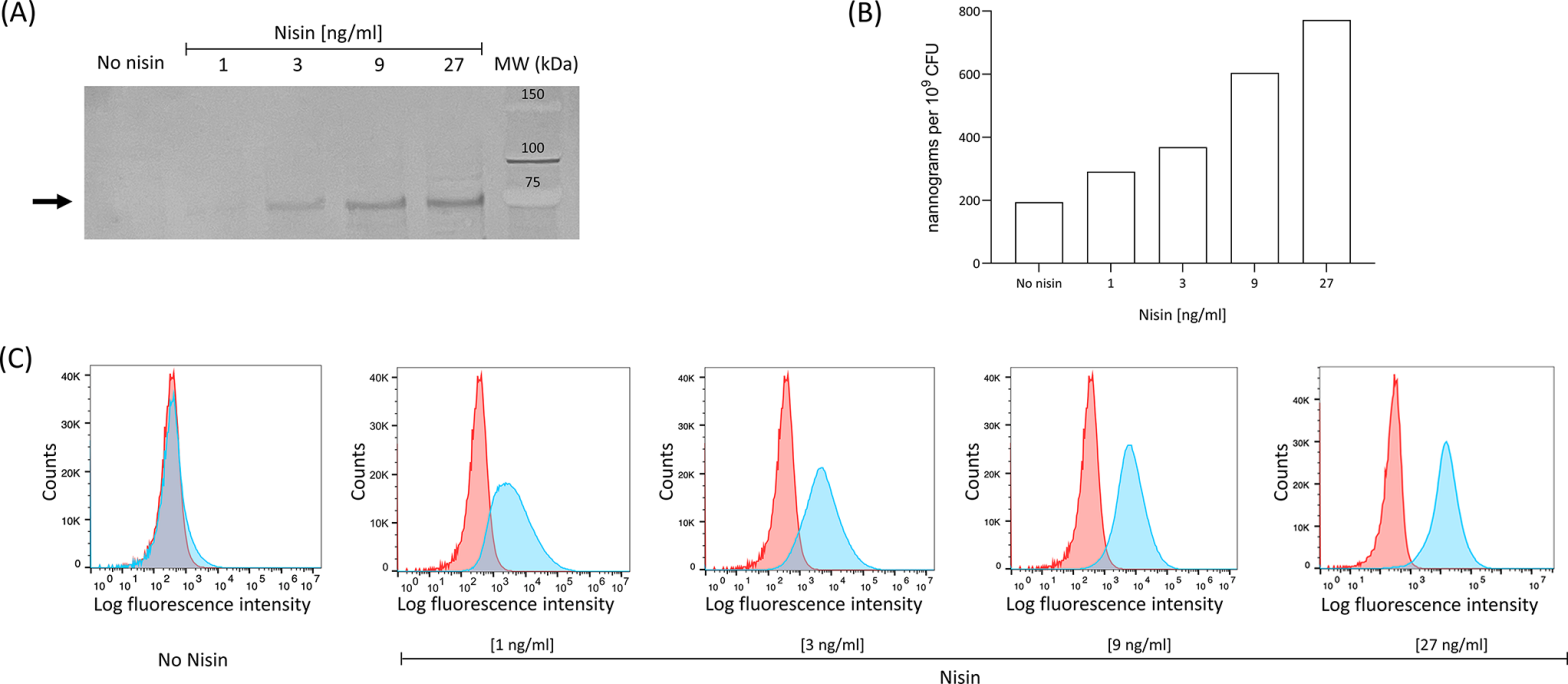
M6-HA1 fusion protein expression analysis in *S. pneumoniae.* M6-HA1 was identified by Western blot (**A**) using FR372 cell extracts from cultures induced with 3-fold serially diluted nisin concentrations. Samples were incubated with anti-M6 rabbit serum and an expected 69 kDa band (indicated by arrow) corresponding to the M6-HA1 protein was found. M6-HA1 was quantified by Dot blot (**B**) using two-fold diluted lysates starting from 1 × 10^7^ CFU. For standard curve building, known quantities of the purified HA1 protein ranging from 25 ng to 195 pg were spotted on the membrane. Flow cytometry analysis of M6-HA1 expression on the *S. pneumoniae* surface was performed using anti-M6 rabbit serum (**C**). The fluorescence peak of the M6-HA1 expressing cells (blue) is shifted to the right compared with control strain (red). Image refers to a representative experiment. Full images of Western blot, Dot blot and the gMFI plot of Flow cytometry analysis reported in Figure S1

**Fig. 5:**
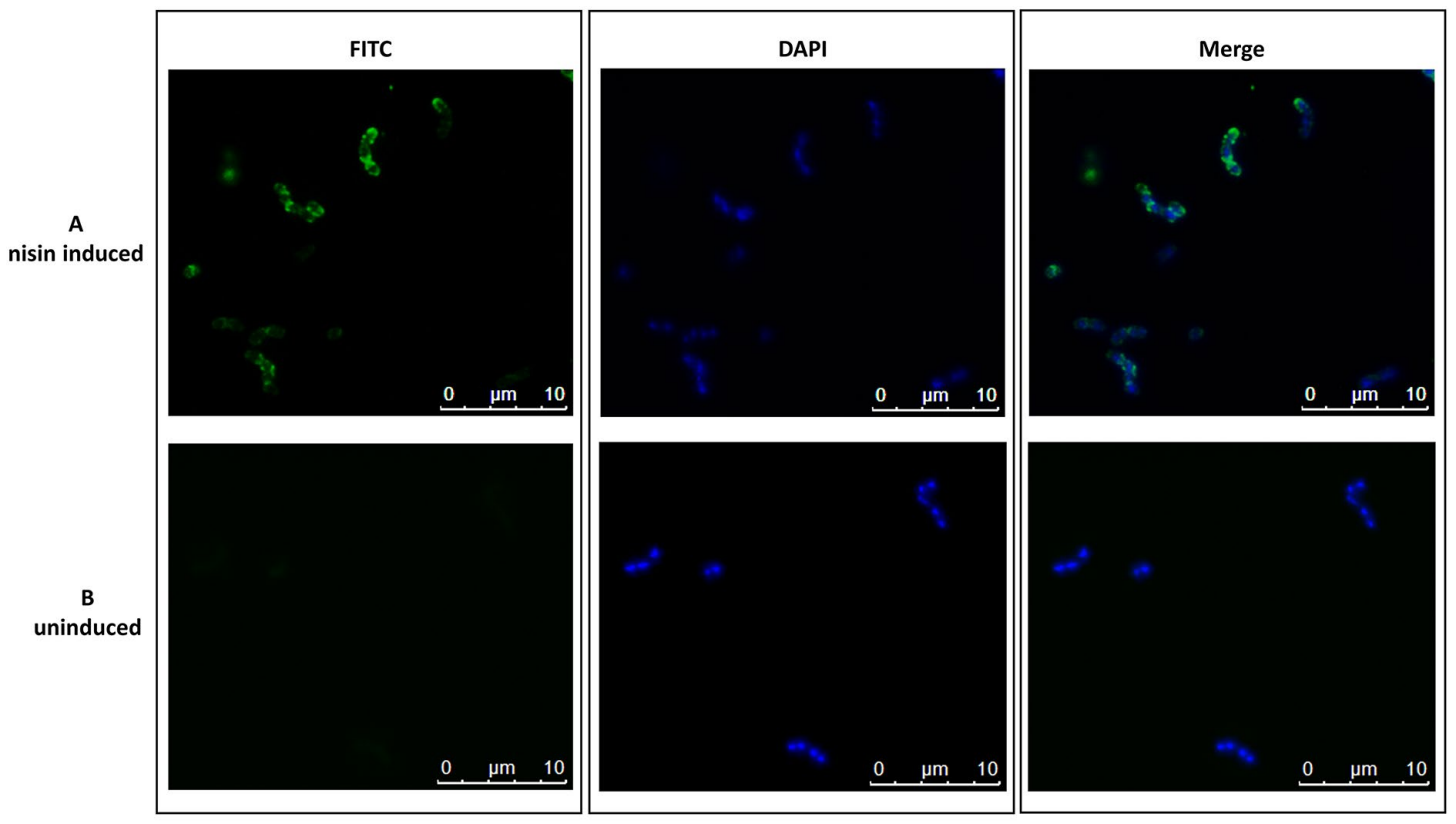
Immunofluorescence microscopy analysis of *S. pneumoniae*. Bacterial cells (approximately 10^6^ CFU) (**A**) induced with 27 ng/ml nisin and (**B**) uninduced, were treated with anti-M6 rabbit serum, then with a FITC-conjugated goat anti-rabbit IgG secondary antibody. Bacteria were counterstained with DAPI II. The left panel shows images acquired in the FITC fluorescence filter, the middle panel shows the DAPI fluorescence filter, while the left panel shows merged images

The expression of M6-HA1 fusion protein was analyzed by Western blot, Dot blot, Flow cytometry and Immunofluorescence microscopy. Western blot analysis was performed on cell extracts of *S. pneumoniae* FR372 cultures induced with a nisin concentration ranging from 1 to 27 ng/ml. An estimated 69 kDa reactive band, corresponding to the predicted M6-HA1 protein size, was detected when using anti-M6 rabbit serum (Fig. 4A). Semi-quantitative analysis of M6-HA1 expression was carried out by Dot blot on cell extracts using an anti-HA1 rabbit monoclonal antibody. M6-HA1 protein quantities ranged from 291 to 772 ng per 10^9^ CFU of induced pneumococcal cultures (Fig. 4B). Flow cytometry analysis performed on live bacterial cells showed the presence of the M6-HA1 protein on the pneumococcal surface (Fig. 4C). The increase in fluorescence intensity observed in FR372 compared with the negative control was correlated with nisin concentration. The presence of the M6-HA1 protein on pneumococcal surface was confirmed by Immunofluorescence microscopy analysis (Fig. 5).

### Mobilization of the Tn*5253*::[*nisRK*]-[*emm6::ha1*] expression system to Streptococci and enterococci

**Fig. 6:**
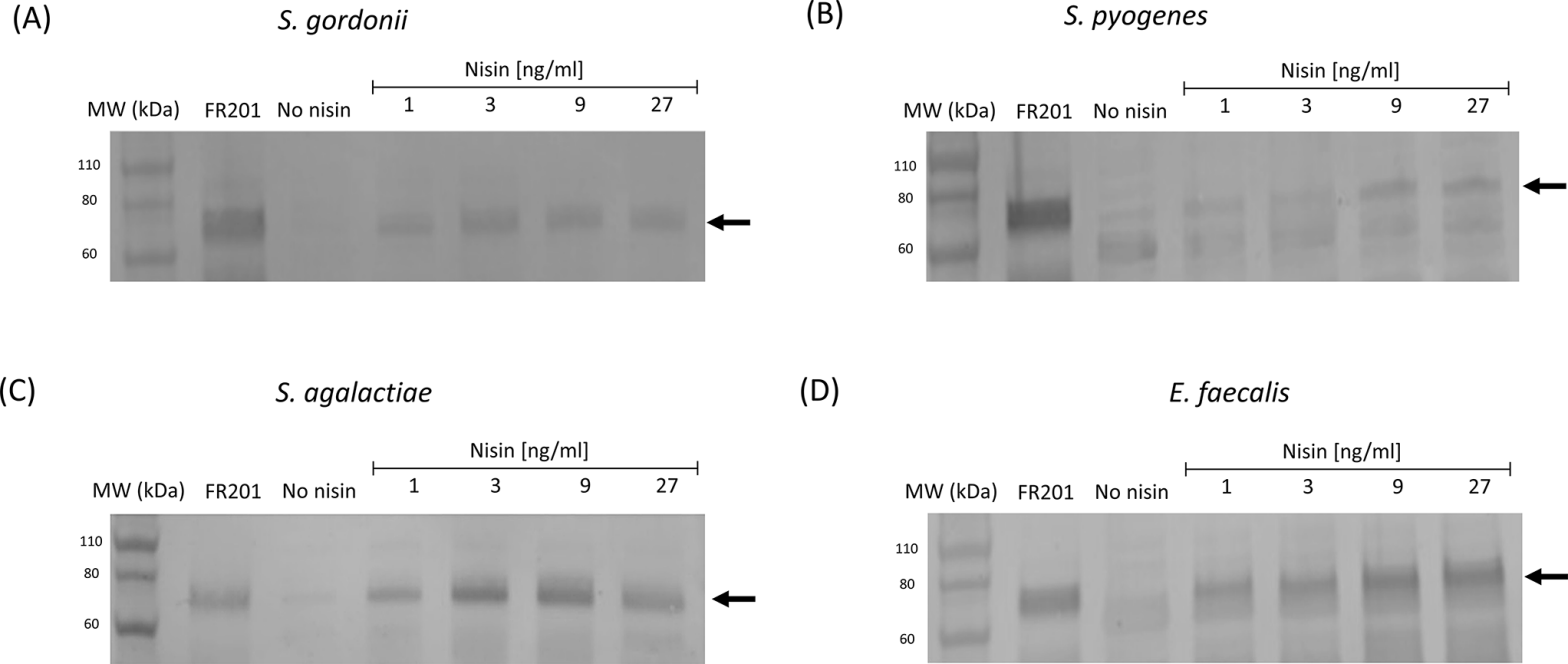
M6-HA1 fusion protein expression analysis in other streptococci and enterococci. M6-HA1 identification was obtained by Western blot carried out on recombinant bacterial cell extracts from cultures induced with 3-fold serially diluted nisin concentrations. Samples were incubated with anti-M6 rabbit serum and an expected 69 kDa band (indicated by arrow) corresponding to the M6-HA1 protein was found. *S. gordonii* FR201 strain, constitutively expressing the M6-HA1 protein, was used as positive control

The recombinant ICE Tn*5253*::[*nisRK*]-[*emm6::ha1*] containing the nisin-inducible expression system was transferred by conjugation in different streptococcal recipients including *Streptococcus gordonii* V288, *Streptococcus pyogenes* SF370, *Streptococcus agalactiae* H36B and in *Enterococcus faecalis* JH2-2 (Table 1). The conjugal transfer yielded transconjugants of each bacterial species, with conjugation frequencies ranging from 8.1 × 10^− 7^ to 2 × 10^− 5^ transconjugants per donor in *E. faecalis* and *S. gordonii*, respectively. Representative transconjugants were tested for genetic stability of the recombinant element, and used for further analysis. The *emm6.1::ha1* transcript was detected and quantified by RT-qPCR (Fig. 3B) and Western blot analysis confirmed the presence of the M6-HA1 protein in all bacterial species (Fig. 6). Furthermore, M6-HA1 protein was quantified using Dot blot analysis, whereas protein surface expression was detected by Flow cytometry. M6-HA1 protein quantities ranged from a minimum of about 290 ng in *S. gordonii* to a maximum of about 600 ng in *E. faecalis* upon induction with 27 ng/ml of nisin (Figure S2). Accordingly, Flow cytometry analysis confirmed the presence of M6-HA1 protein on the cell surfaces in all induced transconjugants with a positive shift slightly increasing in a nisin dose-dependent manner.

## Discussion

In this study we developed and validated a nisin-inducible chromosomal host-vector expression system based on ICE Tn*5253* of *S. pneumoniae* and transferable in a variety of streptococci and enterococci. The system is constituted of a shuttle vector capable of replication in *E. coli* but not in *S. pneumoniae*, where it integrates in Tn*5251*, a Tn*916*-family ICE contained in the composite element Tn*5253*. The insertion vector carries the *L. lactis* two-component regulatory system *nisRK* and a multiple cloning site for target gene insertion under the control of the *L. lactis* nisin inducible promoter P*nisA*. For system validation, we cloned the fusion gene *emm6.1::ha1* encoding the fusion protein containing the signal peptide, the 122 N-terminal and the 140 C-terminal aa of the *Streptococcus pyogenes* M6 surface protein joined to the HA1 subunit of the influenza virus A hemagglutinin. The *emm6.1::ha1* was previously cloned in our *S. gordonii* host-vector system under a strong constitutive promoter with efficient M6-HA1 fusion protein expression on bacterial surface (Iannelli et al., unpublished). Here, we observed a tunable regulation of M6-HA1 protein expression when inducing bacterial cultures with various concentrations of nisin, making this expression system suitable for mucosal vaccine studies requiring fine adjustement of antigen dose. A basal gene expression was found in the untreated control culture as already reported (Eichenbaum et al. 1998). Widely used plasmid-based expression systems are capable of autonomous replication in the host, resulting in multiple plasmid copies that can lead to perturbations of the cell physiology (Friehs 2004). Since Tn*5253* integrates at a single, specific site into the bacterial chromosome (Santoro et al. 2021) and replicates passively during chromosomal duplication (Johnson and Grossman 2015), our ICE-based expression system ensures a single-copy integration of the target gene. Furthermore, plasmids can be lost if selection is not maintained over generations, while ICEs are vertically transmitted to the bacterial daughter cells. In fact, genomic analysis of the population structure of *S. pneumoniae* highlighted that ICEs are conserved within lineage suggesting that a stable integration into the chromosome ensures genetic maintenance of these mobile elements (Croucher et al. 2011). On the other hand, when using integrative vectors for bacterial manipulation, the choice of a transcriptionally neutral chromosomal site represents a major drawback (Guiral et al. 2006b; Keller et al. 2019). Our genetic system allows to overcome this limitation by using the natural interaction between Tn*5253* and its bacterial host. Tn*5253* integration site is located in the conserved essential *rbgA* gene (Santoro et al. 2021), thus the expression system has the potential to transfer to a wide range of bacterial species. In fact, the system was efficiently transferred by conjugation in different streptococcal species including *S. gordonii*, *S. pyogenes*, *S. agalactiae* and *E. faecalis*, where a nisin dose-response relationship was also detected. Tn*916*, a broad host range conjugative transposon, was previously used for the construction of ICE-based host-vector expression systems for pathogenic, non transformable bacteria (Mullany et al. 1994, 2012; Manganelli et al. 1998; McBride and Sonenshein 2011a, b). Tn*5251* is highly homologous to Tn*916* and generally transfers ‘hitchhiking’ Tn*5253* conjugation machinery, but it is also capable of independent conjugal transfer in different bacterial species (Santoro et al. 2010). This characteristic further expands the potential bacterial host range where the Tn*5253*-based nisin-inducible expression system can be used. In conclusion, we constructed a transferable ICE Tn*5253*-based host-vector system tested for controlled heterologous gene expression and potentially usable for a wide range of applications including complementation assays and/or physiological studies in *S. pneumoniae* and other transformable and non transformable bacteria including pathogenic species otherwise difficult to manipulate.

## Electronic supplementary material

Below is the link to the electronic supplementary material.

Supplementary Material 1

Supplementary Material 2

## Data Availability

All data supporting the findings of this study are available within the paper and the supplementary materials. All images in this study were created by the authors and are original designs.
